# Permanent Dipole Moment in a Quantum-Confined Two-Dimensional Metal Revealed by Electric Double Layer Gating

**DOI:** 10.1021/acs.nanolett.5c00500

**Published:** 2025-03-25

**Authors:** Nader Sawtarie, Jonathon R. Schrecengost, Krishnan Mekkanamkulam Ananthanarayanan, Nithil Harris Manimaran, Shubham Sukumar Awate, Chengye Dong, Ke Xu, Yuanxi Wang, Joshua A. Robinson, Noel C. Giebink, Susan K. Fullerton-Shirey

**Affiliations:** † Department of Chemical and Petroleum Engineering, University of Pittsburgh, Pittsburgh, Pennsylvania 15260, United States; ‡ Department of Materials Science and Engineering, The Pennsylvania State University, University Park, Pennsylvania 16802, United States; ¶ Microsystems Engineering, Rochester Institute of Technology, Rochester, New York 14623, United States; § School of Physics and Astronomy, Rochester Institute of Technology, Rochester, New York 14623, United States; ∥ Department of Physics, University of North Texas, Denton, Texas 76203, United States; ⊥ Department of Chemistry, The Pennsylvania State University, University Park, Pennsylvania 16802, United States; # Department of Physics, The Pennsylvania State University, University Park, Pennsylvania 16802, United States; @ Department of Electrical Engineering, The Pennsylvania State University, University Park, Pennsylvania 16802, United States; △ Department of Electrical Engineering and Computer Science, University of Michigan, Ann Arbor, Michigan 48109, United States; ∇ Department of Electrical and Computer Engineering, University of Pittsburgh, Pittsburgh, Pennsylvania 15260, United States

**Keywords:** 2D metal, electrolyte gating, confinement heteroepitaxy, Stark shift, electro-optic

## Abstract

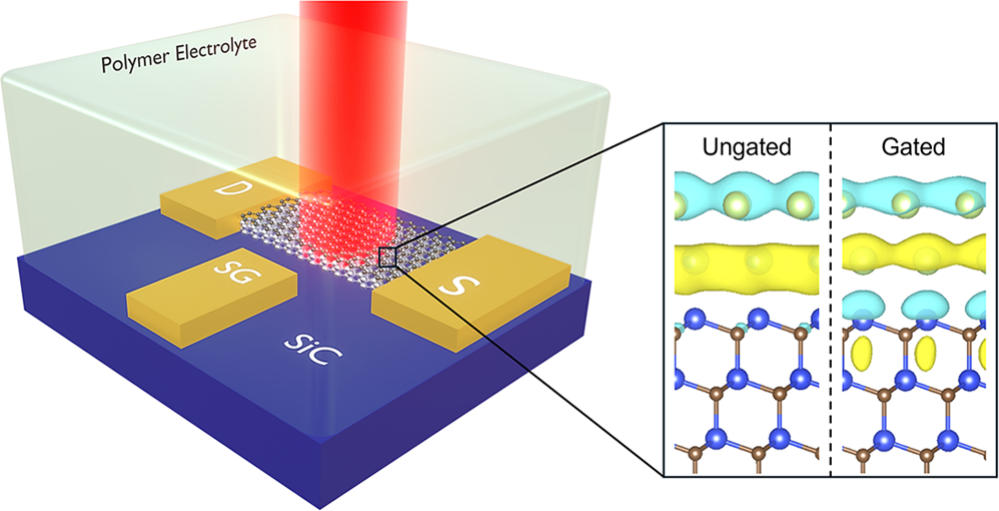

The tunable optical properties of metals through size-dependent quantum effects have attracted attention due to synthesis of chemically stable, ultrathin, and two-dimensional metals. Gate tunability, from the reduced screening of low-dimensional metals, adds an additional route for control over optical properties. Here, two-dimensional (2D) Ga is synthesized via confinement heteroepitaxy and patterned into electric-double-layer (EDL) gated transistors. 2D Ga is predicted to have an out-of-plane permanent dipole moment resulting from a non-centrosymmetric interface. Alternating current EDL gating induces a measurable change in 2D Ga reflectivity of Δ*R*/*R* ∼ 8 × 10^–4^. The optical response is dominated by a linear Stark shift of 1.8 meV, corresponding to a 0.4 D change in the permanent dipole moment between the ground and excited states of 2D Ga. These results are the first demonstration of 2D metal gating and the first direct evidence of a permanent dipole moment in a 2D metal.

When metals are scaled from bulk to thicknesses less than 10 nanometers, size-dependent quantum effects emerge.^1^ Such ultrathin metals have optical properties that distinguish them from bulk; for example, the Kerr susceptibility of ultrathin Au is 4 orders of magnitude larger than that of bulk.^2^ Driven by confinement, the enhanced plasmonics of ultrathin metals^3^ can be leveraged for optoelectronic applications such as biosensors,^4^ photothermal therapy,^5,6^ photodetectors,^7^ and photovoltaics.^8,9^ Further scaling reduces ultrathin metals to the ultimate two-dimensional (2D) limit (i.e., one-three atomic layers); however, reports on such materials are limited mainly by the challenge of synthesizing chemically stable 2D metals. Instability arises from the large number of undercoordinated surface atoms, restricting reports of 2D metals to noble metals.^10−15^ Moreover, quantum confinement can increase the density of states (DOS) near the Fermi level, which increases chemical reactivity^16^ and therefore air instability.

Recently, confinement heteroepitaxy (CHet) has enabled the growth of *air-stable* 2D metals including non-noble metals. In CHet, metals including Ga, In, or Ag intercalate between SiC and epitaxial graphene (EG), yielding atomically thin, epitaxial metal films that are typically one to three monolayers thick.^17^ Crucially, the EG overlayer protects the metal from environmental oxidation;^18^ thus, CHet offers a foundational platform for electrical and optical characterization of atomically thin 2D metals. Moreover, such extreme scaling and chemical stability opens the possibility of a 2D metal with properties that can be tuned by field effect, a level of control that is not accessible with bulk metals.

Field-effect control of the optical response of ultrathin metal films^19^ and graphene^20−22^ has been demonstrated previously using electric double layer (EDL) gating. This technique replaces the gate oxide with an electrolyte, a material that conducts ions, but not electrons/holes. When a gate bias is applied, ions accumulate at the gate/electrolyte and channel/electrolyte interfaces and induce image charge. The proximity of ions and induced charge (∼1 nm) create capacitance densities up to 10 μF cm^–1^,^23^ which is an order of magnitude larger than oxide dielectrics. Such charge densities arise from the large electric field (∼V nm^–1^), which correspond to sheet carrier densities on the order of 10^14^–10^15^ cm^–1^.^24^ For a metal, even one that is atomically thin, such large electric fields are required to modulate charge because the intrinsically high charge density will screen a weak field.

EDL gating of ultrathin Au has been demonstrated to induce a change in the Drude contribution to the dielectric function that can alter measurable quantities such as reflectivity and the surface plasmon dispersion.^19^ CHet-grown 2D metals are unique by comparison because, in addition to their Drude response, they also feature quantum-confined interband transitions in the visible frequency range,^25^ leading to resonance in this range. Optical activity in the visible range further differentiates CHet-grown 2D metals from other plasmonic materials, including graphene and ultrathin metals, with optical responses predominantly in the infrared and near-infrared (NIR) region.^26−28^ Such a quantum-confined interband transition in the visible frequency range, combined with the unusual fixed dipole moment that is predicted to exist in CHet-grown 2D metals due to their variation in covalent to metallic to van der Waals bonding character,^17^ suggests the possibility of a richer electro-optic response beyond plasmonic effects induced by changes in carrier concentration.

Here, we develop a low-frequency, alternating current (AC) EDL gating method to measure the electroreflectance (ER) of 2D Ga in the NIR to visible range. To the best of our knowledge, this is the first demonstration of the gating of an atomically thin, 2D metal. We find that the optical response of 2D Ga is dominated by a linear Stark shift of the quantum-confined interband transition in 2D Ga, indicating the presence of a permanent dipole moment in the system and an ∼0.4 D difference between the ground and excited states. This observation is in good agreement with density functional theory (DFT) calculations, which reveal an inverted permanent dipole moment and thus provide both experimental and theoretical support for the polar nature of 2D Ga. Further, the linear Stark shift of 1.8 meV estimated by optical modeling of the experimental data is consistent with the Stark shift from DFT.

Two atomically thin layers of Ga are intercalated between epitaxially grown and lithographically patterned EG and the underlying SiC substrate through CHet. Ga is introduced primarily at the patterned EG edges, as opposed to defects as originally described for unpatterned EG by Briggs et al.^17^ Such edge-initiated selective area intercalation makes possible the patterning of various 2D Ga geometries up to hundreds of micrometers. 2D Ga/EG device fabrication details and characterization data are available in Section 1.2 of the SI and Figure S1, respectively.

A cross-sectional scanning-transmission electron microscopy (STEM) image of a device shows the atomic layers of EG and 2D Ga and confirms a Ga bilayer (Figure 1(a)). DFT calculations predict thermodynamically stability of one to three layers of 2D Ga,^29^ which combined with ellipsometry measurements show that electronic and optical properties vary with thickness due to changes in the band structure.^25^ The atomic structure predicted by DFT is shown in Figure 1(b), highlighting the variation in interlayer spacing that arises from changes in bond type: covalent to metallic to van der Waals. This non-centrosymmetric bonding character spanning <1 nm is responsible for the record-breaking nonlinear optical properties of 2D Ga^30^ and is predicted to give rise to a permanent ground state dipole moment.^25^

**Figure 1:**
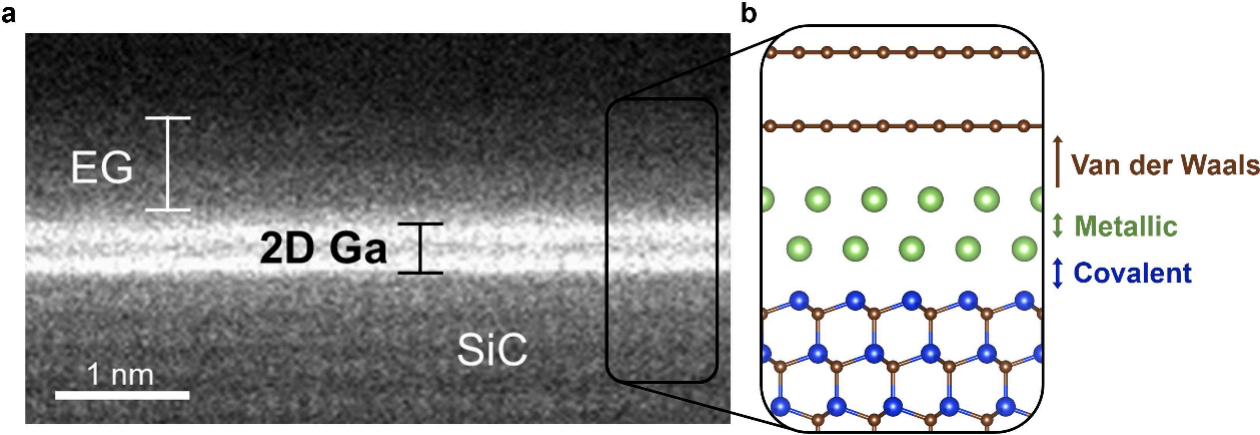
Atomic structure of 2D Ga measured by STEM and predicted by DFT. (a) STEM image of a cross section of a 2D Ga electro-optic device showing bilayer 2D Ga intercalated between a 6H SiC substrate (0001 facet) and bilayer EG, indicating a 2D Ga thickness of ∼0.5 nm, consistent with previous reports.^17,25^ (b) Output of DFT calculations showing the predicted atomic structure of bilayer 2D Ga. Variation in interlayer spacing highlights the non-centrosymmetric bonding.

After device fabrication, poly(ethylene oxide) (PEO) and CsClO_4_, with an ether oxygen to Cs^+^ molar ratio of 20:1, were deposited from solution, covering the entire device. An optical image of a PEO:CsClO_4_-covered EG field-effect transistor (FET) used to optimize AC EDL gating is shown in Figure 2(a), where side gates permit optical access to the channel. As depicted in Figure 2(b), when a positive side gate bias is applied, a cationic EDL consisting of Cs^+^ will form at the interface between the electrolyte and the channel. Reversing the polarity will drift ClO_4_^–^ ions to the channel surface.

**Figure 2:**
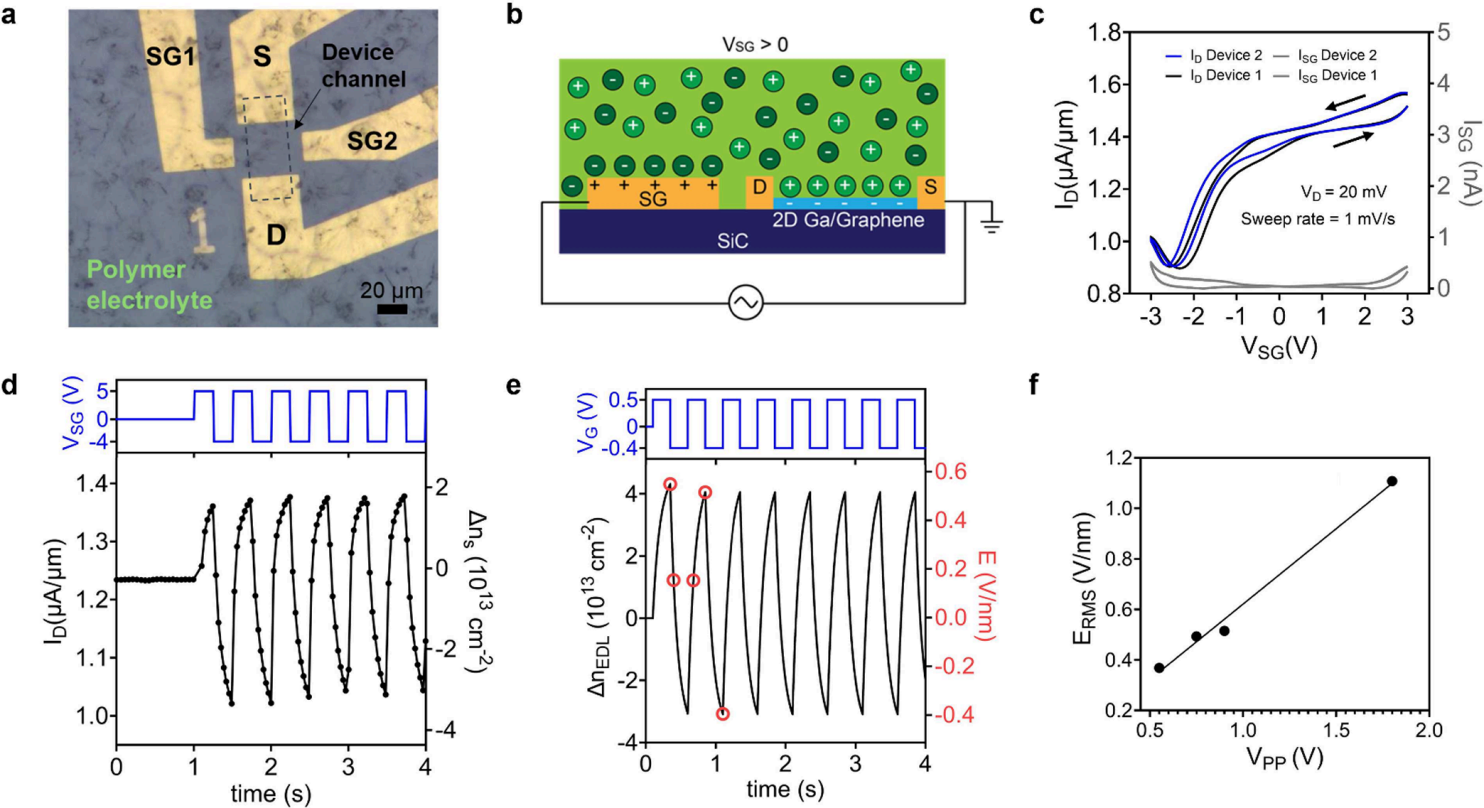
EDL gating of the 2D Ga/EG FETs. (a) Optical image of an EG FET (i.e., without 2D Ga) with 10 μm of polymer electrolyte, PEO:CsClO_4_, used to optimize the AC EDL gating technique. (b) Schematic of AC EDL gating for which a cationic double layer is formed at the interface between the polymer electrolyte and the 2D Ga/EG channel. (c) Double sweep transfer measurements of two 2D Ga/EG electro-optic devices; *V*_D_ = 20 mV with a sweep rate of 1 mV/s. (d) Asymmetric square wave bias of +5 to −4 V applied to the 2D Ga/EG FET at 2 Hz and resulting current density induced by AC EDL gating. The corresponding change in sheet carrier density, Δ*n*_s_, is indicated on the secondary *y*-axis. (e) Output of COMSOL modeling of a parallel plate capacitor for which an asymmetric square wave bias of +0.5 to −0.4 V is applied at 2 Hz and the resulting change in Δ*n*_EDL_ (black line) and electric field strength (red open circles). (f) Linear relationship between *E*_RMS_ and peak-to-peak gate bias, *V*_pp_, from COMSOL.

Gate control of the channel is demonstrated by the double-sweep transfer characteristics in Figure 2(c). Current flows through both EG and 2D Ga, as indicated by two features: (1) a minimum conduction current associated with the Dirac point of EG, and (2) a 3-fold increase in current density compared to EG FETs grown in the same growth chamber as this work.^31^ The additional charge carriers provided by 2D Ga are also reflected in the 3-fold increase in the output characteristics compared to EG alone (Figure S2(a)) and in Hall-effect measurements of 2D Ga/EG (Figure S2(d)) that show an order of magnitude increase in sheet carrier density under electrolyte gating compared to EG alone (Figure S2(b)). Such an increase in charge carriers upon 2D Ga intercalation is consistent with a decrease in electrical resistance that would be expected to accompany conduction through a metal.

Because 2D Ga is only two atomic layers thick, changes in microreflectivity will be extremely small; therefore, an oscillating electric field is applied, and a lock-in amplifier is used to isolate and amplify the signal. To apply such an oscillating field, AC EDL gating was developed. The frequency at which EDLs can form and dissipate in an electrolyte depends on the mobility of the ions, which can range from microseconds for liquids to seconds for solid polymer electrolytes.^31,32^ Thus, frequency modulation on the order of a few Hz is achievable for PEO-based electrolytes at room temperature. Furthermore, gate voltages are applied for shorter times in AC EDL gating than in a conventional gate, meaning that partially formed double layers are generated within each cycle. Therefore, larger gate voltages can be used to accelerate EDL formation rates without inducing Faradaic current.^31^

AC EDL gating was accomplished by connecting a function generator to a side gate electrode (see Figure S3). With *V*_D_ = 20 mV, a 2 Hz square-wave was applied with a peak-to-peak bias (*V*_pp_) of 9 V and an offset bias (*V*_offs_) of 0.5 V. That is, *V*_SG_ is varied from +5 to −4 V, as shown in Figure 2(d) along with the resulting *I*_D_. The need for a voltage offset relates to differences in cation and anionic mobility in the electrolyte. Because cations interact more strongly with the polymer backbone than anions, they tend to have a lower mobility in PEO.^31^ Thus, in the absence of a voltage offset, the channel current modulation will drift with time due to the differences in ion mobility (see Figure S4(a)). Devices that experience such current drift eventually fail, likely due to the buildup of one EDL over the other, presumably the anionic EDL.

The change in *I*_D_ induced by gating is assumed to result from a change in the total carrier concentration of the channel (i.e., EG plus 2D Ga). To estimate the change in sheet carrier density, Δ*n*_s_, of the FET used for electro-optic measurements, Hall bar devices were fabricated from the same batch of 2D Ga/EG so that a comparable contact resistance could be assumed. As detailed in Section 4 of the SI, a relationship is established between the sheet carrier density measured by Hall effect and the sheet resistance, *R*_s_, in the FET used for electro-optic measurements. Using this relationship, AC EDL gating of the FET is found to induce a Δ*n*_s_ of ∼5 × 10^13^ cm^–2^, as shown on the secondary *y*-axis of Figure 2(d).

Next, we estimate the root-mean-square electric field strength, *E*_RMS_ (V/nm), induced by AC EDL gating. As sketched in Figure 2(b), the measured change in sheet carrier density is assumed to be equal to and opposite the concentration of ions in the EDL, *n*_EDL_. COMSOL modeling is used to approximate the electric field that is generated from the experimentally measured charge carrier densities. With details of the modeling given in Section 4 of the SI and Figure S5, the electric field strength varies from −0.4 to 0.5 V/nm with 2 Hz AC EDL gating and a peak-to-peak gate bias, *V*_pp_, of 0.9 V, which is plotted on the secondary *y*-axis of Figure 2(e). COMSOL modeling confirms the partial EDL formation that is expected with AC gating. *E*_RMS_ is also modeled for various *V*_pp_ and plotted in Figure 2(f), revealing a linear relationship.

To connect the COMSOL modeling back to the experiment, the *E*_RMS_ of the experiment was estimated as follows. First, Δ*E*/Δ*n*_EDL_ was calculated from COMSOL, as shown in Figure S6 in the SI. Along with the experimentally measured Δ*n*_s_ (secondary *y*-axis of Figure 2(d)), this slope was used to estimate the electric field strength induced by AC EDL gating during the ER measurements. Thus, for an experimentally measured Δ*n*_RMS_ of 1.7 × 10^13^ cm^–2^, the *E*_RMS_ is estimated as 0.22 V/nm. Using an intrinsic sheet carrier density (*n*_0_) of 8.1 × 10^13^ cm^–2^, also measured by Hall effect, the modulation of the free carrier density, Δ*n*_RMS_/*n*_0_, is calculated as ∼20.7%.

To understand how the strong gate field and EDL-induced change in free carrier density affect the optical response of 2D Ga, ER measurements are carried out using the experimental setup shown in Figure 3(a). Using AC EDL gating, we lock in to the signal at the voltage modulation frequency and synchronously detect fractional changes in the reflected light intensity as small as Δ*R*/*R* < 5 × 10^–5^.

**Figure 3:**
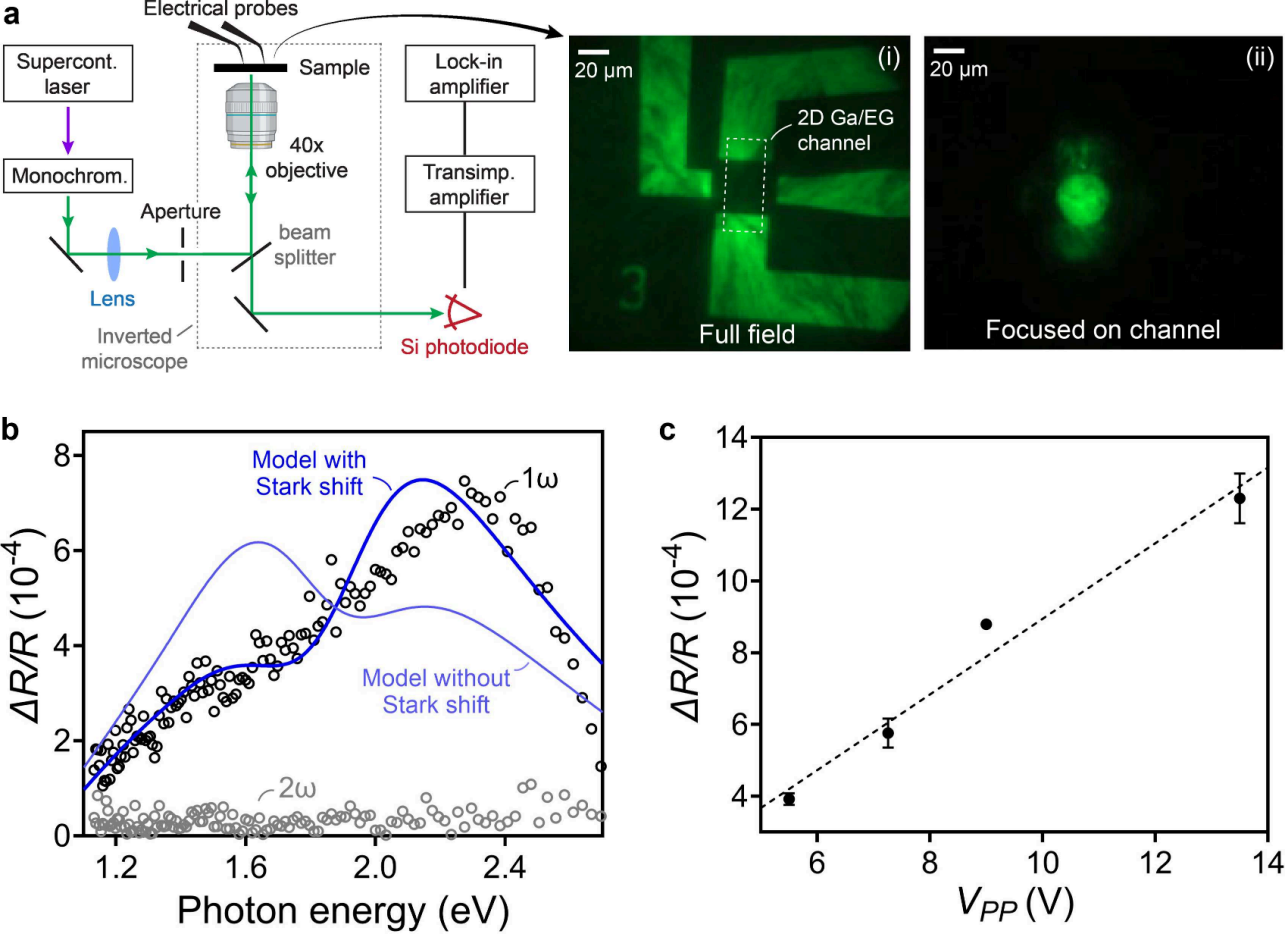
ER measurements of 2D Ga. (a) Experimental setup for measuring ER of AC EDL gated 2D Ga. A supercontinuum laser is monochromated and focused onto the sample using an inverted microscope. Reflected light is recollected and detected synchronously at the modulation frequency by using a Si photodiode and lock-in amplifier. The right-hand images show monochromatic green light (i) illuminating the entire field of view and focused on the channel of the device (ii). (b) ER spectrum was recorded at the first (1ω) and second (2ω) harmonics of the modulation frequency for an AC EDL-gated device under the same drive conditions as in Figure 2(d). The thick blue line is calculated via the transfer matrix model (TMM) assuming a 1.8 meV shift in the energy of the quantum-confined interband transition (Stark shift) located at ∼2 eV, while the thin blue line shows the TMM without including a Stark shift. Full details of the optical model are provided in Section 5 of the SI. (c) Magnitude of the 1ω ER signal at 2.3 eV as a function of the peak-to-peak AC voltage, *V*_PP_. Error bars indicate one standard deviation from the mean from four different measurements.

Figure 3(b) shows the ER spectrum of a 2D Ga device operated under the same AC driving conditions as those in Figure 2(d). A peak at ∼2.3 eV is observed in the first-harmonic signal, while nothing is detected at the second harmonic, which implies that the ER scales linearly with *V*_pp_. Further evidence follows from the linear scaling of *E*_RMS_ with *V*_pp_ shown in Figure 2(f) and of Δ*R*/*R*_1ω_ with *V*_pp_ shown in Figure 3(c). These results were reproduced across several devices (see Figure S7). Control experiments carried out at different spots adjacent to the channel (i.e., SiC substrate and electrolyte) and on other EG-only devices confirm that the ER signal originates from the 2D Ga and not the EG overlayer, SiC substrate, or electrolyte.

Some portion of the ER response should derive from the associated change in the Drude contribution to the 2D Ga optical constants due to the change in the plasma frequency. However, the Drude contribution is strongest in the NIR of the spectrum,^25^ whereas the ER peaks in the visible (see Figure 3(b)) close to a quantum-confined interband transition in 2D Ga.^25^ This observation raises the possibility that the ER is instead due to a Stark shift. To disentangle these two effects, we carry out transfer matrix modeling (TMM) of the differential reflectivity using the ellipsometry model established by Nisi et al.,^25^ which is further described in Figures S8 and S9 as well as Section 5 of the SI. By perturbing parameters such as the plasma frequency of the Drude term in the 2D Ga dielectric function or the energy of the oscillator modeling its interband transition, the impact of each effect on Δ*R*/*R* can be evaluated (see Figure S9). Notably, an order of magnitude lower value for the change in free carrier concentration (i.e., 2% versus the ∼20% measured experimentally) was required to fit the ER data in the lower energy region (<2.0 eV). The large discrepancy between experimentally measured and optically modeled changes in free carrier concentration may be the result of the parameters in the Drude model being unphysical to begin with. The Drude parameters of the model were chosen to fit the data with no electric field, which is shown in Figure S8. Furthermore, the changes in EG and 2D Ga free carrier concentration for TMM needed to be equal and opposite, which raises an intriguing possibility of charge balancing between EG and 2D Ga, but remains an outstanding question.

The results of the modeling are shown as solid lines in Figure 3(b), and they indicate that a ∼1.8 meV shift in the interband oscillator energy is necessary to reproduce the observed ER peak at ∼2.3 eV. Specifically, the model fits with and without the Stark shift are highlighted. From a perturbation theory standpoint, a linear Stark shift arises from a difference in permanent dipole moment between the ground and excited states of a transition (Δμ), whereas a quadratic shift reflects a difference in their polarizabilities.^33^ The linearity of the ER response established in Figure 3(b,c) implies that the contribution from Δμ is dominant and therefore that the energy shift depends on the applied field (*F*) according to Δ*E* = – Δμ·***F***. Taking the *E*_RMS_ strength associated with the first harmonic of the square wave gate voltage (*F*_RMS_ ≈ 0.22 V/nm, which is responsible for the first-harmonic ER signal) and Δ*E* ≈ 1.8 meV, the magnitude of Δμ is approximately 0.4 D.

To understand the microscopic origin of the Stark shift, DFT calculations are used to model the optical response of the system to a static, out-of-plane applied electric field. Figure 4(a) shows the imaginary part of the in-plane dielectric function calculated for applied field strengths in the range of −0.1 to +0.1 V/Å, focusing on the peak of the transition at ∼2 eV, highlighted in the inset. As the field sweeps from negative to positive (blue to red, corresponding to toward and away from the SiC substrate, respectively), this peak shifts linearly by ∼10 meV (see Figure 4(b)). Scaled to the same 0.22 V/nm field strength as the experimental measurement in Figure 3(b), this calculation predicts a 1.7 meV energy shift relative to the zero-field case, which is in close agreement with the experimental value of 1.8 meV.

**Figure 4:**
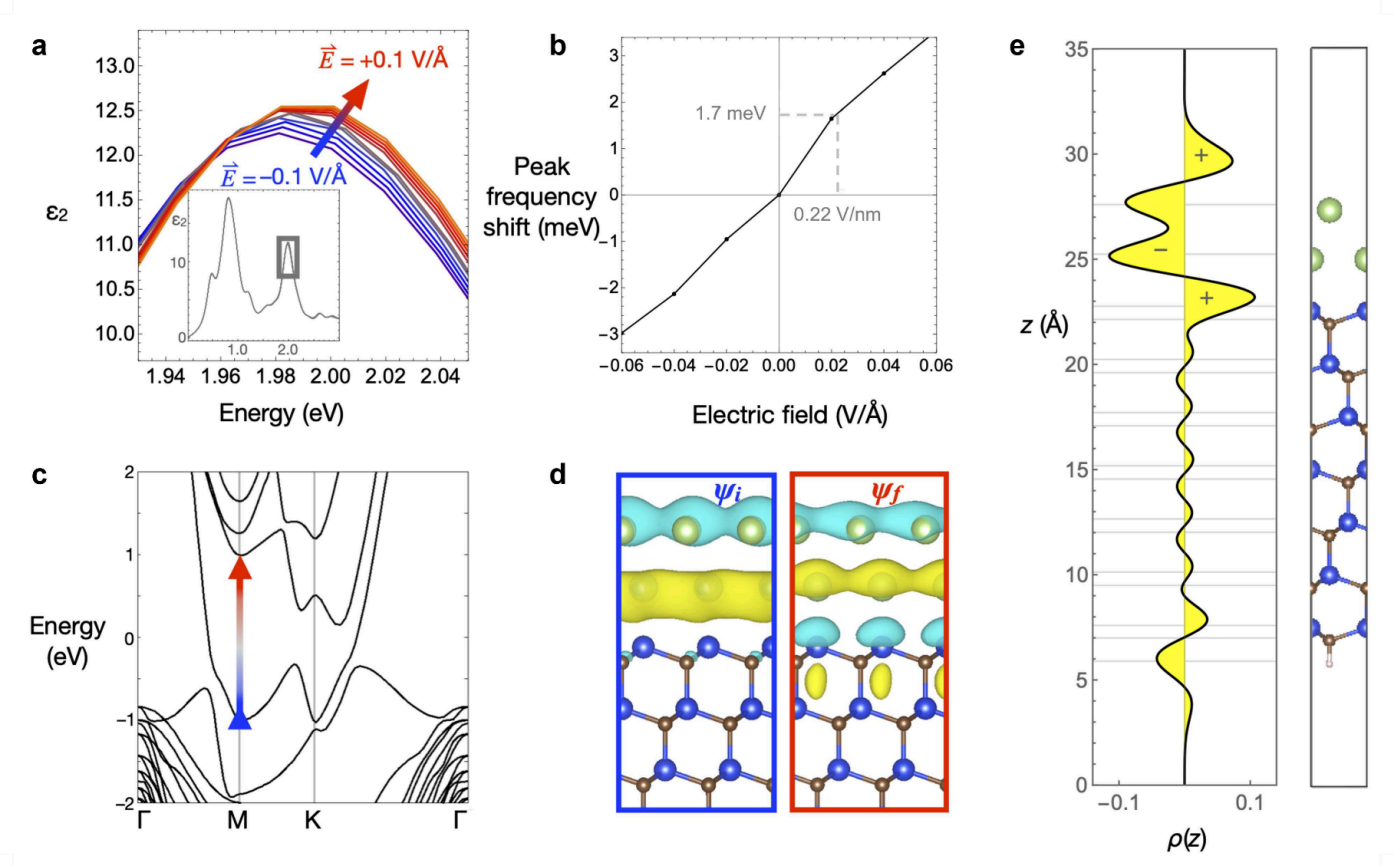
DFT calculations for gated 2D Ga. (a) Imaginary part of the in-plane dielectric function, ϵ_2_(ω), computed for bilayer Ga on a SiC substrate as a function of applied electric field normal to the surface. The main panel shows a close-up of the absorption peak near 2 eV in the inset. (b) Energy shift of this peak as a function of the applied electric field. (c) Band structure of bilayer Ga on SiC, highlighting the interband transition that underlies the 2 eV absorption peak. (d) Wave functions of the initial and final single-particle states involved in the optical transition, using the same red/blue color coding as panel (c). (e) *x*–*y* averaged charge density of the bilayer Ga/SiC slab model as a function of *z* position, aligned with the atomic positions in the model shown at the right, revealing an inverted permanent dipole moment in 2D Ga. The *z*-dependent charge density distribution is obtained by Gaussian smearing of the electronic and ionic charge in the 3D model with broadening parameters of σ = 1.2 and σ = 1.26 Å, respectively.

According to the model, the absorption peak at 2.0 eV originates primarily from interband transitions centered around the M point, as indicated by the arrow in Figure 4(c) connecting an initial (blue) and a final (red) single-particle state. Inspecting the wave functions of these two states in Figure 4(d), we find that this excitation relocates electron density from the Ga to the uppermost layer of Si atoms in the SiC substrate, which produces a change in vertical dipole moment that leads to the observed Stark shift. To determine the direction of the permanent dipole moment of the Ga/SiC system in its ground state, we average the charge density over the *x*–*y* plane as a function of *z* by smearing out both the electronic charge densities and ionic point charges to remove high spatial frequency variation. The result is plotted along *z* in Figure 4(e), revealing an upward-pointing dipole moment at the Ga surface and a larger downward-pointing dipole moment at the Ga/SiC interface. This is unusual for a metal because the dipole moment points inward rather than outward from the surface, which may be useful for catalyzing certain types of chemical reactions.^34,35^

In summary, 2D Ga is synthesized via selective-area CHet and fabricated into electro-optical devices for microreflectivity measurements under EDL gating. To measure the small signals associated with a metal that is only two atomic layers thick, AC EDL gating was developed to generate a large (0.22 V/nm) oscillating electric field, and a lock-in amplifier was used to detect fractional changes in the ER response. The large field strength induces changes in the free carrier concentration of approximately 20%, verified through a combination of Hall measurements and COMSOL modeling. The significant modulation in free carrier concentration induces a measurable change in ER due to plasma-frequency modulation, particularly in the NIR region. However, these measurements reveal that the ER response is dominated by a linear Stark shift in the energy of a quantum-confined interband transition at ∼2.0 eV in 2D Ga that arises from an ∼0.4 D difference in the permanent dipole moment between its ground and excited states. DFT modeling agrees with this observation, providing strong evidence for the existence of an unusual, inward-pointing permanent dipole moment in 2D Ga. This study represents the first experimental measurements of a gated 2D metal and of a permanent dipole in a 2D metal.

## Supporting Information Available

Experimental and computational methods; physical, chemical, electrical, and optical characterization of 2D Ga/EG devices; estimation of electric field strength; optical transfer matrix modeling (PDF)
