# Supplementary material for: Permanent Dipole Moment in a Quantum-Confined Two-Dimensional Metal Revealed by Electric Double Layer Gating
Source: Nano Lett. 2025 Mar 25;25(16):6599–605. doi: 10.1021/acs.nanolett.5c00500 (PMC12023022; doi:10.1021/acs.nanolett.5c00500)
Supplement: Supplementary file 1 — nl5c00500_si_002.pdf [file nl5c00500_si_002.pdf]

Supporting Information: Permanent dipole moment in a quantum confined two-dimensional metal revealed by electric double layer gating

Nader Sawtarie,[†] Jonathon R. Schrecengost,[‡] Krishnan Mekkanamkulam Ananthanarayanan,[‡] Nithil H. Manimaran,[¶] Shubham Sukumar Awate,[†] Chengye Dong,[‡] Ke Xu,[§,¶] Yuanxi Wang,[∥] Joshua A. Robinson,[‡,⊥,#] Noel C. Giebink,[*,@,△] and Susan Fullerton-Shirey[*,†,∇]

†*Department of Chemical and Petroleum Engineering, University of Pittsburgh, Pittsburgh, PA 15260, U.S.A.*

‡*Department of Materials Science and Engineering, The Pennsylvania State University, University Park, PA 14802, U.S.A.*

¶*Microsystems Engineering, Rochester Institute of Technology, Rochester, NY 14623, U.S.A.*

§*School of Physics and Astronomy, Rochester Institute of Technology, Rochester, NY 14623, U.S.A.*

∥*Department of Physics, University of North Texas, Denton, TX 76203, U.S.A.*

⊥*Department of Chemistry, The Pennsylvania State University, University Park, PA 14802, U.S.A.*

#*Department of Physics, The Pennsylvania State University, University Park, PA 14802, U.S.A.*

@*Department of Electrical Engineering, The Pennsylvania State University, University Park, PA 14802, U.S.A*

△*Department of Electrical Engineering and Computer Science, University of Michigan, Ann Arbor, MI 48109, U.S.A*

∇*Department of Electrical and Computer Engineering, University of Pittsburgh, Pittsburgh, PA 15260, U.S.A.*

E-mail: ngiebink@umich.edu; fullerton@pitt.edu

1 Methods

1.1 Epitaxial graphene growth on SiC.

Epitaxial graphene (EG) was synthesized on semi-insulating 6H SiC (Cohenrent Inc) substrate via a two step process. First, the SiC wafer was annealed at 1400 °C for 30 min at 700 torr under 450 sccm Ar and 50 sccm H_2 to remove oxides from the wafer surface. Next, EG was thermally grown on the 0001 plane of 6H-SiC by sublimating Si at 1800 °C and 700 torr with an Ar flow (500 sccm) for 20 min.

1.2 2D Ga intercalation and electro-optic device fabrication.

Photolithography and dry etching was used to pattern the EG for the 2D Ga/EG electro-optic devices. The photoresist SPR 3012 (Shipley) was spin coated onto the surface (4000 rpm for 45 s) and baked on a hotplate for 60 s at 105 °C. Maskless photolithography (MLA 150, Heidelberg) was used to pattern the EG using a dose of 200 mJ/cm2 (λ = 365 nm). The sample was developed using CD-26 (Shipley) for 75 s. EG was dry etched via plasma (ULvac NE 550) with N_2 used as the plasma gas (200 W for 15 s). Lift-off of the photoresist, SPR 3012, was then performed by soaking in PRS-3000 at 60 °C (JT Baker) and IPA, each for 10 min.

2D Ga was intercalated via CHet through the edges of the lithographically patterned EG creating 30 $\times$ 40 μm channels. A custom alumina crucible (Robocasting Enterprises) and a horizontal tube furnace (Thermo Scientific, Lindberg Blue M) with a 1-inch outer diameter quartz tube was used for CHet. The crucible and quartz tube were cleaned in advance by annealing in the furnace at 1000 °C under 200 sccm Ar flow at a pressure of 50 torr for 60 minutes. The crucible was loaded with $\approx$ 50 mg of metallic Ga (purity 99.999%, Sigma Aldrich) with the patterned EG sample facing downwards directly over the Ga precursor. The crucible was then heated to 800 °C (50 °C/min) under 50 sccm Ar flow at a pressure of 500 torr for 30 minutes.

Source/drain and side gate contacts were patterned on the 2D Ga/EG devices using MLA with the same dosage as above. LOR 5A photoresist (JT Baker) was spin coated at 4000 rpm for 45 s and baked for 180 s at 180 °C. The pattern was developed with CD-26 (Shipley) for 60 s. Next, a gentle plasma treatment was performed for 45 s to improve metal contact to the EG surface (Tepla M4L; 50 sccm of O_2 and 50 sccm of He at 50 W and 500 mTorr). Ti/Au contacts and side gate electrodes (5/50 nm)was evaporated (Temescal e-beam evaporator) at a pressure of 1×10^{-7} torr. Photoresist was lifted off with PRS-3000 at 60 °C for 10 min and additional 10 min soak in IPA.

1.3 Electrolyte preparation and deposition.

The polymer electrolyte, PEO:$CsClO_4$, was prepared at a concentration of 10 wt% and ether oxygen to Cs ion ratio of 20:1 by co-dissolving 200 mg of polyethylene oxide (PEO, 99.9% purity, MW = 99,000 g/mol, Polymer Standard Services) and 40 mg $CsClO_4^-$ salt (99.9% purity, Sigma Aldrich) in 2.3 g anhydrous acetonitrile (Sigma Aldrich) inside an Ar-filled glovebox. To create a 10 μm thick electrolyte film, approximately 50 μL was drop cast onto the devices, with the device probe pads protected by tape to keep them electrolyte free for wire bonding. Acetonitrile was allowed to naturally evaporate in the glovebox for $\sim$ 30 minutes, resulting in a solid electrolyte film. The electrolyte thickness is 10 μm to mitigate thin film interference and make it optically transparent in the NIR to visible range.

1.4 Wire bonding.

Sample chips were adhered to a chip carrier (Evergreen semiconductor) using Norland 68 ultraviolet-curable adhesive (cured with UV light λ = 365 for 10 min.) Wire bonds were made with a WestBond wire bonder with an ultrasonic time of 30 ms at a power of 400 W used for both bonds. A low force setting was used for the bond to sample chip, and a high force setting was used for the bond the connection to chip carrier.

1.5 Raman and AFM Device characterization.

2D Ga coverage was evaluated by Raman spectroscopy (Horiba LabRAM HR) using a 532 nm ULF laser with a 1 μm diameter spot size and 300 g/mm grating. For the Raman map shown in Fig. S1(e), an exposure time of 5 seconds with one accumulation was used. Surface topology of the devices was assessed using atomic force microscopy (Bruker Icon AFM) in an Ar environment using Peakforce tapping mode and a ScanAsyst-Air (0.4 N/m) tip.

1.6 Transfer measurements.

A Lakeshore cryogenic probe station (CRX-VF) with a Keysight B1500A semiconductor parameter analyzer was used to make double-sweep transfer measurements. The temperature of the sample stage was held at 300 K under 5×10^{-7} Torr. V_{SG} was held constant for 600 s to provide sufficient time for the ions to reach steady state before starting the measurement. V_{SG} was swept between +3 and –3 V with a sweep rate of 1 mV/s and V_D of 20 mV. Leakage current, I_{SG}, was monitored during the transfer measurement.

1.7 Hall measurements.

Hall bar devices were patterned using the same MLA process as described above and measured using a Keysight B1500A semiconductor parameter analyzer in a Lakeshore CRX-VF probe station with a variable superconducting magnet with a maximum field strength of 2.5 T. The sample stage temperature was maintained at 300 K and 5×10^{-7} Torr. Prior to each measurement, a constant V_{SG} was applied for 900 seconds to allow the ions to reach steady state. A constant current of 50 μA was applied through the 2D Ga/EG channel. The Hall voltage was monitored by gradually increasing the magnetic field from 0 to $+1.5 \pm 0.01$ T. This sequence was repeated with the polarity of the magnetic field reversed to -1.5 ± 0.01 T. To eliminate the effect of geometric asymmetry, the Hall voltages at 0 T were subtracted from all the Hall voltages to $B = \pm 1.5$ T. This measurement procedure was repeated for all

the reported V_{SG} values. The Hall coefficient, $R_H = V_{12eff}/(I_D B) = 1/(qn_s)$ in cm^2/C, was calculated using the effective Hall voltage V_{12eff}, magnetic field B, and elementary charge, q.

1.8 COMSOL Modeling.

Ion transport in response to an AC electric field was modeled in COMSOL using a metal-electrolyte-metal configuration where the solid electrolyte was 0.5 × 0.5 μm (Fig. S5(a)). The modified Poisson-Nernst-Planck (mPNP) equation was used.

$$\frac{\partial c_\pm}{\partial t} - \nabla \left(D_\pm \nabla c_\pm + D_\pm \frac{q}{k_b T} z_\pm c_\pm \nabla V + \gamma \right) = 0$$

where $c_\pm, D_\pm$, and $z_\pm$ are the local ion concentrations (ions/cm^3), ionic diffusivities (cm^2/s), and charge numbers of cations and anions. q (C) and $k_b T$ (J) are the elementary charge and thermal energy respectively. Poisson's equation, $\nabla \cdot (-\varepsilon\varepsilon_0 \nabla V) = q\,(c_+ - c_-)$, was used to solve for the local potential, V; γ represents the steric repulsion factor to account for the ions' physical size. Derived by Kilic et al.,[1] the steric factor is defined as

$$\gamma = D_\pm c_\pm a^3 \frac{\nabla\,(c_+ - c_-)}{1 - a_+^3 c_+ - a_-^3 c_-}$$

where a_+ and a_- are the diameters of the cation and the anion, respectively. The ion diameters and diffusivities were set equal to 4 $\mathring{A}$ and 10^{-12} cm^2/s, respectively.[2,3] The permittivity (ε) of the electrolyte was set to 10.[4]

1.9 Electro-optic measurements.

Chip carrier-mounted samples were placed on the deck of an inverted microscope with the electrolyte facing down. A source-measure unit (SMU) was used to apply a 20 mV DC bias and measure the current between the source and drain electrodes while the side gate was driven by an AC voltage provided by a function generator. Illumination was provided by a supercontinuum laser filtered through a monochromator, resulting in output light with a

full-width at half-maximum spectral bandwidth of ~ 5 nm. This light was focused through the microscope on to the device (incident through the electrolyte) using a 40× objective. The reflection was recollected through a 50/50 beam splitter, detected on a Si photodiode, and converted to a voltage with a transimpedance amplifier. The AC component of this voltage was isolated with a lock-in amplifier referenced to the function generator while the DC component was measured with a multimeter. Dividing the former by the latter yields the differential reflectivity, $\Delta R/R$.

1.10 Density Functional Theory.

All DFT calculations were performed using the Vienna Ab-initio Simulation Package (VASP) with the Perdew-Burke-Ernzerhof (PBE) parametrization of the generalized gradient approximation exchange-correlation functional and projector augmented wave pseudopotentials.[5] Plane-wave expansion cutoff energies were 500 eV. k-point sampling densities were set to $21 \times 21 \times 1$ for accurate placement of the Fermi level. All structures were relaxed until atomic forces converge within 0.01 eV/Å. All self-consistency calculations were performed including an out-of-plane dipole correction that compensates for the intrinsic dipole moments of the modeled structure.[6]

1.11 Scanning Transmission Electron Microscopy of 2D Ga/EG channels.

Cross-sectional samples were prepared via focused ion beam (FIB) milling (Helios Nanofab Dualbeam 660, FEI Inc.) An amorphous carbon with a thickness of 400 nm was predeposited via e-beam then ion beam before FIB milling. Ga^+ ion beam starting at 30 kV was used for milling and gradually reduced to 1 kV for thinning. High-resolution STEM of the cross-sectional sample was achieved in a FEI dual aberration-corrected Titan3 G2 60–300 S/TEM at 200 kV with a beam current of 70 pA.

2 2D Ga/Epitaxial Graphene Device Characterization

Device fabrication is described in Section 1.2 of the SI; here, topological characterization with Atomic Force Microscopy (AFM) and chemical characterization with Raman spectroscopy are detailed. Fig. S1(b) is a topology scan of a 2D Ga/epitaxial graphene (EG) electro-optic device (before electrolyte deposition), with the side gate (SG) and channel regions labeled. The terracing in the AFM scan is from SiC substrate, with step edges of 3–5 nm. Importantly, the SiC terraces are oriented parallel to channel, thereby reducing the possibility of a discontinuity in 2D Ga/EG channel via the sizable SiC step edge. It is known that such step edges can cause a break in 2D metal films intercalated via CHet.[7]

A topological scan of a 1×1 μm region of the 2D Ga/EG channel of the same device is shown in Fig. S1(c). The root mean square roughness increases from 0.15 to 0.25 nm after lithography, attributed to photoresist residue from the lithography process, which is typical for patterning EG.[8] Minimizing photoresist residue is critical for effective EDL gating - our previous study showed that removing the residue with AFM cleaning can increase channel current in transition metal dichalcogenide FETs by $\approx 250\%$.[9]

Raman spectra of the 2D Ga/EG channel are shown in Fig. S1(d) in the ultra low frequency (ULF) region at 70 equally spaced points in channel (blue data), and 28 equally spaced points in the etched EG (i.e., bare SiC) regions just outside the 2D Ga/EG channel (black data). Peaks at ≈ 25 cm^{-1} and 55 cm^{-1} provide evidence for the presence of Ga (phase II).[10] Furthermore, the bare SiC shows no ULF signal, indicating the successful etching of EG. The isolation of 2D Ga/EG is essential to prevent a short between the channel and side gate.

A Raman intensity map of the ULF spectra in the range of 20–30 cm^{-1} for the bare SiC and the 2D Ga/EG channel is shown in Fig. S1(e). 2D Ga is only present in the channel region as indicated by the blue pixels, and crucially, not present in the etched region thereby providing isolation of side gate and channel. Fig. S1(f) shows Raman spectra of the 2D Ga/EG channel (blue data) in the 1300 to 3000 cm^{-1} range with the SiC substrate

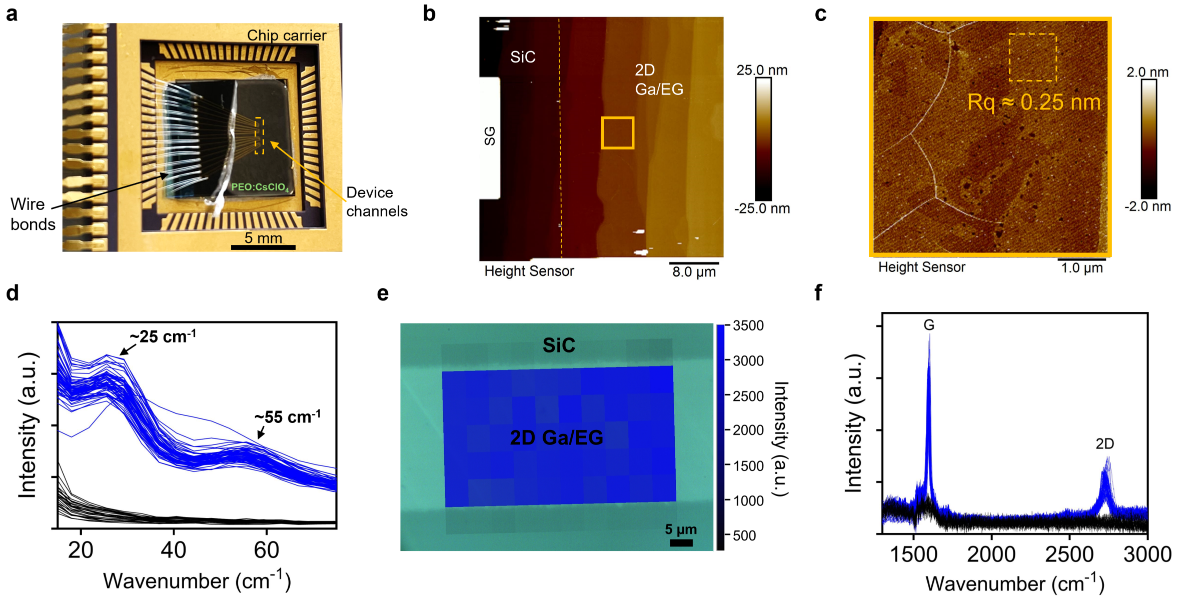

Figure S1: 2D Ga/EG device characterization (a) EG control devices with polymer electrolyte, PEO:$CsClO_4$, mounted on chip carrier with wire bonding for making electrical connections in inverted microscope set up as shown in Fig. 3(a) in the main text and Fig. S3 in SI. A 5 mm gap was kept between devices and probe pads so that a 40x microscope objective could be focused on device channels without interfering with wire bonds (b) AFM topology scan of the 2D Ga/EG electro-optic device (before electrolyte deposition); SiC and 2D Ga/EG regions are separated by vertical dashed line. The yellow box indicates the 1×1 μm zoomed area in (c). Root-mean-squared surface roughness is ≈ 0.25 nm in zoomed area.(d) Raman spectra in ULF region of the 2D Ga/EG channel with characteristic 2D Ga (phase II) ULF peaks at ≈ 25 cm^{-1} and 55 cm^{-1} (blue data). Locations outside of the channel (i.e., bare SiC) are black data. (e) Raman intensity map in the range of $20 - 30$ cm $^{-1}$, corresponding to the peak intensity of 2D Ga ULF peak at ≈ 25 cm^{-1}. Blue pixels in the channel region indicate presence of 2D Ga (phase II) and grey pixels in the etched EG region indicate lack of 2D Ga (i.e., bare SiC). (f) Raman spectra of the 2D Ga/EG in the 1300 to 3000 cm^{-1} range with 2D peak FWHM of 50–60 cm^{-1} indicating bilayer EG (blue data). Etched EG (i.e., bare SiC) has no G or 2D peaks (black data).

background signal subtracted. G and 2D peaks indicate the presence of EG, and a full width half max (FWHM) of 50–60 cm^{-1} suggests the presence of bilayer EG[11]. The black data correspond to regions of etched EG (i.e., bare SiC), and the lack of G and 2D peaks indicate successful channel isolation.

3 2D Ga/EG Device Electrical and Optical Characterization

The output characteristics of 2D Ga/EG electro-optic devices measured for this study are shown in Fig. S2(a). With V_S grounded, V_D was swept from +0.2 to –0.2 V and the resulting current indicated Ohmic contact between the Au source/drain electrodes and 2D Ga/EG channel. Furthermore, there is no measurable hysteresis, and all the 2D Ga/EG devices have approximately the same output characteristics, indicating electrical similarity between devices. The EG control device also showed Ohmic contact, but with approximately a third the current of 2D Ga/EG devices.

To quantify and compare changes in sheet carrier density after 2D Ga intercalation of EG, sheet carrier density versus V_{SG}, measured via Hall-effect, were made on EG only and 2D Ga/EG devices. Details of the these measurements are described above in Section 1.7 of the SI. The average sheet carrier density, n_s, as a function of V_{SG} is shown in Fig. S2(b) and (d) for EG only and 2D Ga/EG devices respectively. The standard deviation in Hall voltage, V_{12}, at B = 0 and ±1 T was propagated through each calculation step, and the final uncertainty in the n_s is reported as error bars. These measurements reveal an order of magnitude larger sheet carrier density under electrolyte gating for the 2D Ga/EG devices (Fig. S2(d)) versus EG only devices (Fig. S2(b)), providing strong evidence that a significant portion of charge carriers is from 2D Ga. In addition, transfer measurements of an electrolyte gated 2D Ga/EG FET with channel width and length of 37 × 7 μm are shown in Fig. S2(c).

The experimental setup to measure the electroreflectivity (ER) spectrum of 2D Ga is

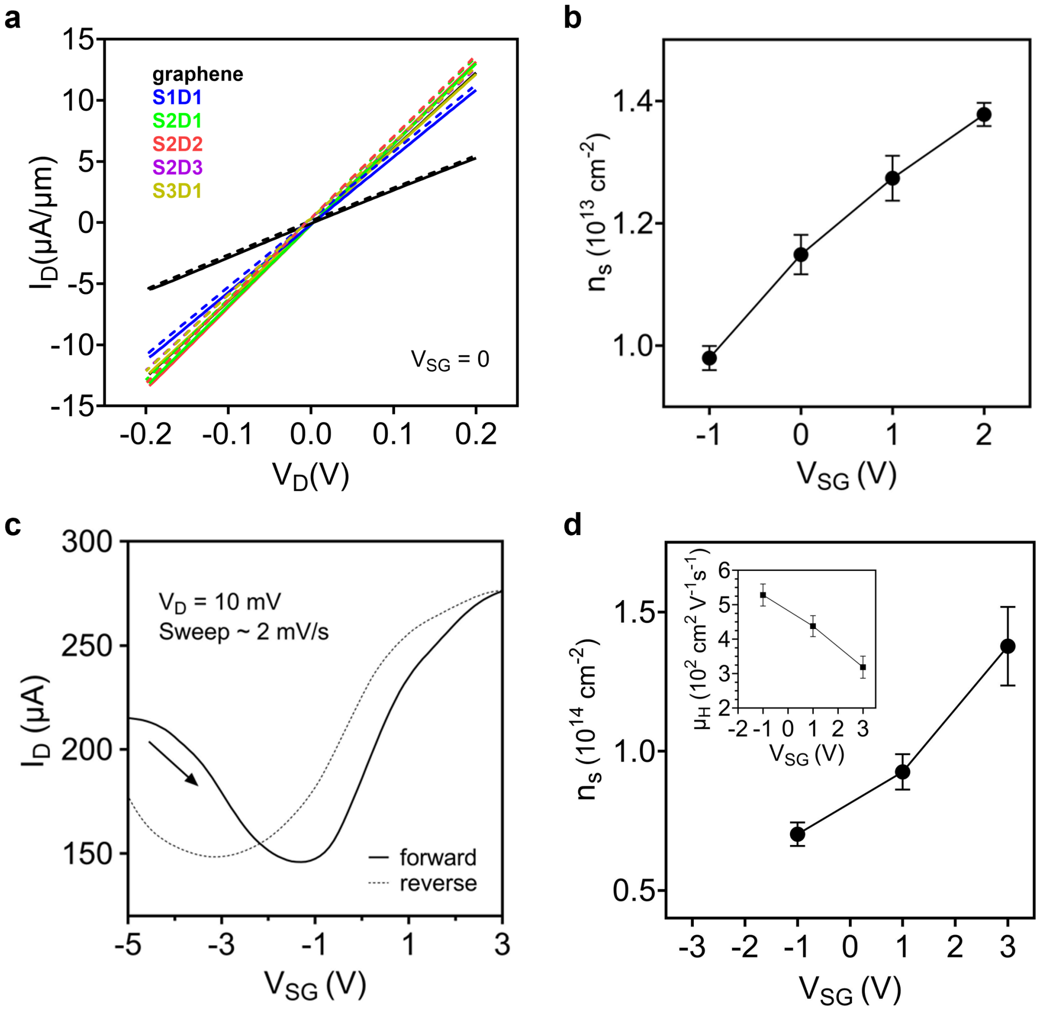

Figure S2: Electrical characterization of EG only and 2D Ga/EG devices (a) Output characteristics of EG control devices (labeled "graphene") and 2D Ga/EG electro-optic devices. Forward sweeps are solid and reverse are dashed. $V_{SG} = 0$ and sweep rate for $V_D = 60$ mV/s (b) Sheet carrier density versus V_{SG} of an EDL-gated EG control device measured by Hall-effect (c) Transfer measurements of 2D Ga/EG device (W = 37 μm, L = 7 μm) from same batch as the 2D Ga/EG electro-optic devices reported in the main manuscript; $V_D = 10$ mV and sweep rate = 2 mV/s (d) Sheet carrier density versus V_{SG} of an EDL-gated 2D Ga/EG device measured by Hall-effect on the same sample chip as FET measured in part (c). Inset shows corresponding mobility (μ_H) from the same Hall measurements.

shown in Fig. S3. A supercontinuum laser is filtered through a monochromator and focused into a fiber optic cable, where it is then recollimated and focused into an inverted microscope onto the 2D Ga sample channel area. This light is then recollected through reflection through a beam splitter, and detected by a photodiode and lock-in amplifier. A front aperture restricts the incident light to only illuminate the 2D Ga/EG channel. Source measurement unit (SMU) 1 applies V_D = 20 mV while a function generator applies an AC V_{SG} = [+5 V, –4 V]. The AC EDL gated modulated reflectivity signal (ΔR) is measured via a lock-in amplifier synced to the function generator frequency, while the DC reflectivity signal is measured by SMU 3. This process is repeated at different wavelengths taken in 10 nm steps, with the bandwidth of the laser having a FWHM of ≈ 4 nm. A source measure unit (SMU) applies a bias across the channel (source-drain) while a function generator applies an AC bias to the side gate and source.

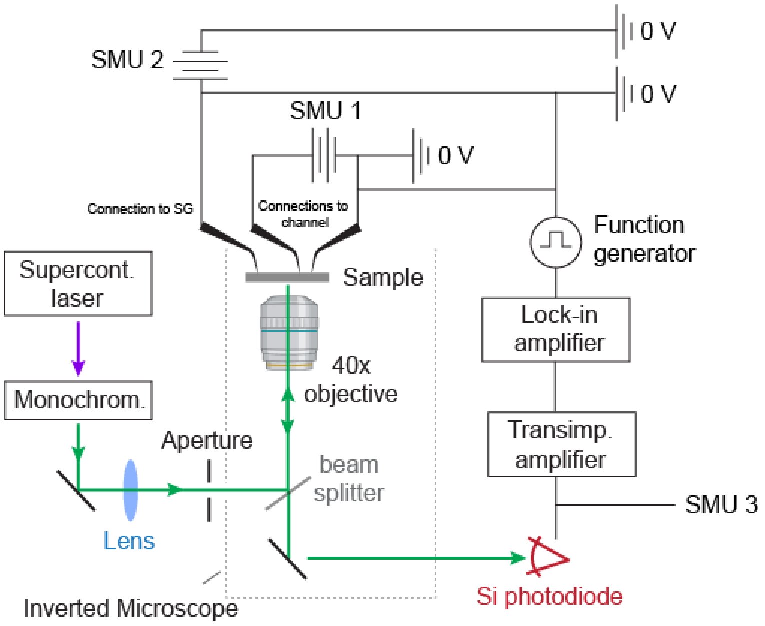

Figure S3: Detailed schematic of electroreflectivity experimental setup.

For the electrolyte gated FETs, the AC EDL gating response is measured for two different voltage protocols and the current versus time is shown in Fig. S4. Fig. S4(a) shows the response of channel current, I_D, when under a symmetric AC square wave bias at a frequency of 2 Hz and with a total magnitude of 9 V (i.e., V_{SG} = [+4.5 V, –4.5 V]). Note that V_{pp}

corresponds to the side gate voltage peak to peak range and V_{offs} corresponds to the amount of offset between positive and negative V_{SG}. As shown in Fig. S4(a) there is a downward drift in channel current with a symmetric V_{SG}, the reasons for which are discussed in the following paragraph. Fig. S4(b) shows the response of I_D when under an asymmetric square wave bias with V_{offs}= 0.5 (i.e., V_{SG} = [+5 V, –4 V]) also at a frequency of 2 Hz. The lack of drift in channel current indicates balanced anionic and cationic EDL formation.

One possible explanation for the asymmetry in the channel current response from different polarities of V_{SG} is the difference in the mobility of cations (Cs^+) and anions (ClO_4^-), where cations have a lower mobility because of coordination with ether oxygen in PEO.[12] Thus, an asymmetric V_{SG} was needed to balance cationic and anionic EDL formation. Unbalanced EDL formation leads to either a drift upward (if cationic EDL formation is faster) or a drift downward (if there is a anionic EDL buildup). In Fig. S4(a) a symmetric gate bias is used which leads to a downward drift of channel current and an anionic EDL buildup. This build up, combined with $V_{SG} > \pm 3$ V, leads to electrochemistry that results in an electrical short and a loss of gate control. It is crucial that anionic and cationic EDL formation is balanced because unbalance will lead to charging of the electrolyte, and high leakage current through the electrolyte which could induce electrochemistry and device failure. To account for the differences in mobility between cations and anions, a positive offset (V_{offs} = 0.5 V) in side gate bias was used. By deploying a slightly asymmetric V_{SG}, the downward drift in channel current was eliminated, as shown in Fig. S4(b). AC EDL gating needed to be stabilized for roughly 30 minutes for microreflectivity measurements over the full visible spectrum. Fig. S4(b) only shows stable AC EDL gating (i.e., without channel drift) over 60 seconds after which the measurement was stopped as a precaution to preserve the device for optical measurements. It should be noted that AC EDL gating measurements were taken first in the electro-optical experimental set up shown in Fig. S3 to demonstrate channel current modulation, after which optical measurements were conducted. Furthermore, only electrical or optical measurements could be taken at a time in the electro-optical experimental set

up. Thus, AC EDL gating measurements over 60 second time periods were taken before and after optical measurements for each device to determine whether cationic and anionic EDL formation was balanced over the duration of the optical measurements. Any channel current drift over the optical measurement would lead to rapid device failure and invalidate the previous optical measurement.

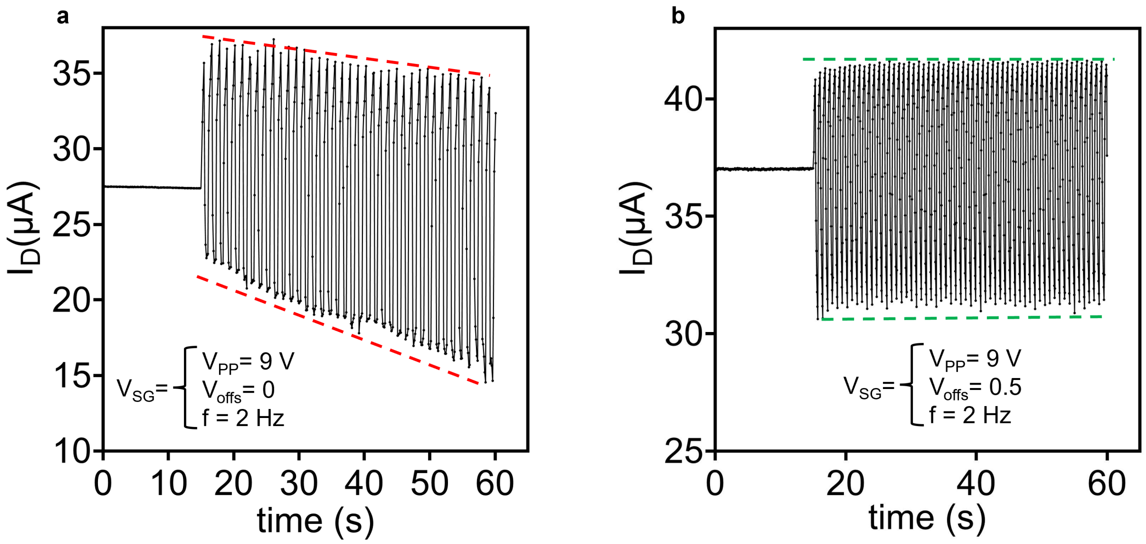

Figure S4: AC EDL gating (a) Channel current I_D versus time under symmetric square wave side gate bias with V_{pp} of 9 V and V_{offs} of 0 and frequency of 2 Hz. (b) Channel current I_D versus time under asymmetric square wave side gate bias with V_{pp} of 9 V and V_{offs} of 0.5 V (i.e., V_{SG} = [+5 V, –4 V] and frequency of 2 Hz.

Notice that the current density range between V_{SG} = +5 and –4 V in Fig. 2(d) in the main text requires a significantly smaller V_{SG} range to access in the corresponding transfer measurements Fig. 2(c) in the main text. The reason is the sluggish mobility of the ions in the polymer. Larger I_D is reached in the transfer measurements of Fig.2(c) because of the slow (1 mV/s) sweep rate compared to the fast sweep rate used in AC gating (40,000 mV/s). In other words, sufficient time is not provided in the AC approach for a full EDL to be established before the polarity is reversed; therefore, the maximum current modulation corresponds to partially formed cationic and anionic EDLs.

4 Estimating the Electric Field Strength by Experiments and COMSOL Modeling

The electric field strength is required to calculate the magnitude of the Stark shift. To determine this value, we use a combination of experiments and COMSOL modeling. Experimentally, we first confirm that the change in sheet resistance, ΔR_s, as a function of gate bias, V_{SG}, is similar on two chips: the one containing the electro-optic devices on which the ER signal is measured, and one containing FETs and Hall bar devices. Note that the 2D Ga was grown on both chips simultaneously, and the same fabrication procedure (described in Section 1.2 of the SI) was used for both. Once confirmed that the two chips share similar transfer characteristics, the change in sheet carrier density, Δn_s, was measured by Hall-effect for the same gate voltage range to establish the relationship between ΔR_s and Δn_s. Finally, COMSOL modeling is used to relate Δn_s to the change in net ion density in the EDL, Δn_{EDL}, and thus the change in electric field strength, ΔE. The details of this process are described below.

Relating ΔV_{SG} to ΔR_s. As shown in Section 3 of the SI, transfer measurements were taken on a 2D Ga/EG FET located on the same chip as Hall bar devices (see Fig. S2 (c)). The maximum channel current, I_D, modulation occurs between V_{SG} = +3 and –1 V, which corresponds to R_s of 0.19 and 0.36 kΩ/□, respectively, for a ΔR_s of 0.17 kΩ/□. This range is comparable to the 2D Ga electro-optic device under AC EDL gating (V_{SG} = [+5 V, –4 V], which has a ΔR_s of 0.13 kΩ/□ (Fig. 2(d)).

Relating ΔR_s to Δn_s. Next, Hall-effect measurements on 2D Ga/EG Hall bars (see Fig. S2(d)) were used to relate change in sheet resistance to change in sheet carrier density. Because the Hall bars were fabricated on the same chip as the FETs, we expect comparable sheet and contact resistances for both devices. In the same V_{SG} range as the FET (i.e., +3 to –1 V), the modulation of n_s in the Hall devices is 0.702×10^{14} to 1.378×10^{14}/cm^2, giving a $\Delta n_s = 6.7 \times 10^{14}$/cm^2. Because the two chips were grown simultaneously and have

comparable transfer characteristics, we can use the relationship between ΔR_s and Δn_s in the FET and Hall bar chip to estimate the Δn_s in the chip that contains the 2D Ga/EG electro-optic devices. Dividing Δn_s by ΔR_s gives a proportionality factor, or k factor, of 3.96 which is used to relate the ΔR_s values from 2D Ga/EG electro-optic devices in response to AC EDL gating to Δn_s (Fig. 2(d), secondary y-axis).

Relating Δn_s to ΔE. As mentioned in the manuscript and sketched in Fig. 2(b) in the main text, the measured change in carrier concentration of the channel is assumed to be equal and opposite the concentration of the EDL formed directly above it. Therefore, the change of sheet carrier density in the channel (Δn_s) is assumed to be equal to the change in net ion density in the EDL (Δn_{EDL}). COMSOL modeling was used to estimate the root-mean-square electric field strength, E_{RMS} (V/nm), based on the net ion sheet density of the EDL. To capture the response of the EDL to an applied AC field, voltage pulses of alternating sign and width of 250 ms were applied to the top gate electrode at a frequency of 2 Hz with the bottom electrode grounded. Positive to negative peak-to-peak biases (V_{pp}) of 0.55, 0.75, 0.9 and 1.8 V were simulated individually, and the voltage pulse train is shown in Fig. S5(b). The corresponding net ion sheet density induced in the EDL, n_{EDL}, is also shown in Fig. S5(b).

To calculate the electric field induced by the EDL, voltage versus distance is plotted at each of the time points indicated by a red circle in Fig. 2(e). As shown in Fig. S5(c), the steep voltage drop across the EDL and the shallow voltage drop into the bulk are each fit to a line, and the EDL thickness, (t_{EDL}), is determined as the location where the two lines intersect. At each time point indicated by a red circle in Fig.2(e), the electric field strength, E, is calculated as the voltage divided by t_{EDL}.

Subsequently, E_{RMS} under AC EDL gating from electro-optics experiments was calculated as follows. First, electric field strength, E (V/nm), was plotted against n_{EDL} (10^{13} ions/cm^2) and a linear relationship was found, which is plotted in Fig. S6. The linear relationship between E (V/nm) and n_{EDL} (10^{13} ions/cm^2) was described by $E = 0.139 \times n_{EDL} - 0.000607$.

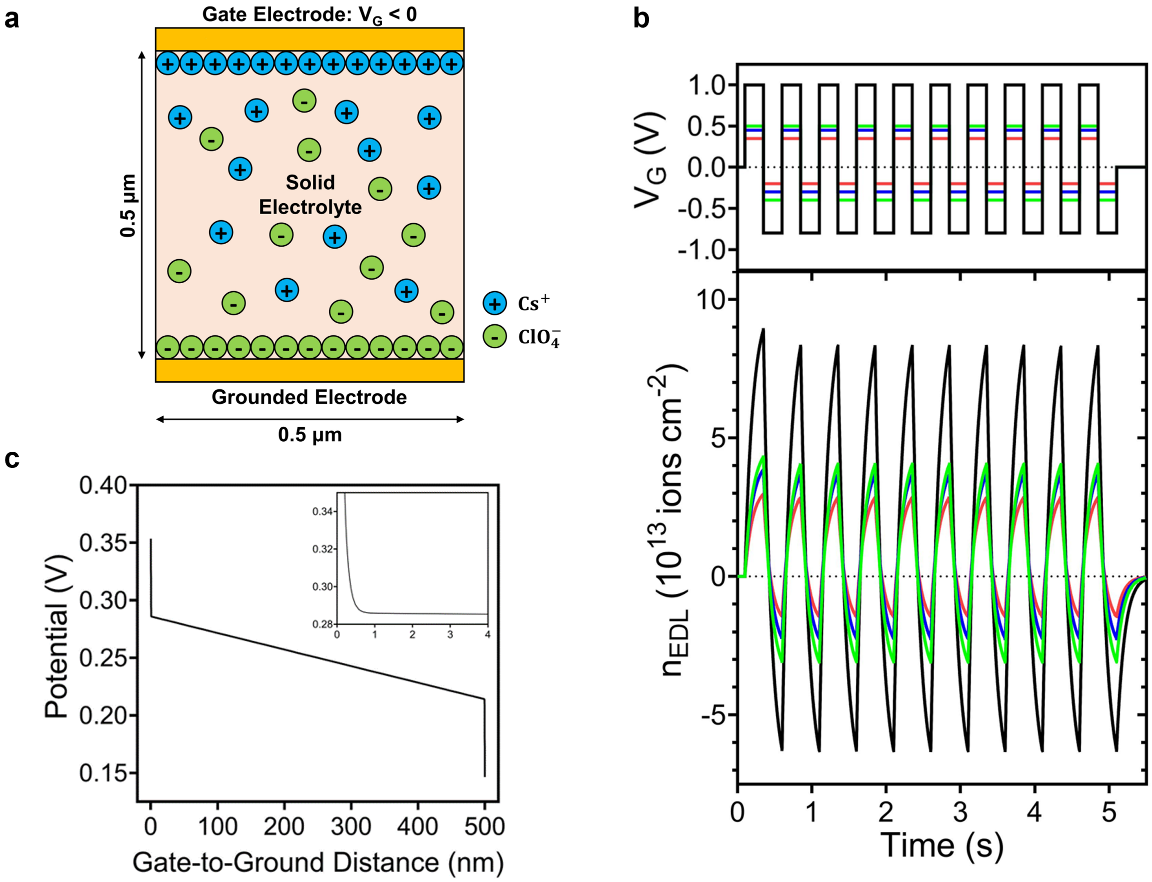

Figure S5: COMSOL modeling to estimate E_{RMS} (a) Schematic of the COMSOL parallel plate capacitor model showing EDLs forming at the electrodes in response to a negative gate bias input (b) Asymmetric square wave pulse train at f = 2 Hz, and its corresponding response in ion sheet density in the EDL, n_{EDL} (10^{13} ions cm^{-2}) (c) Potential drop across the electrolyte (from gate to ground) at t = 0.35 s (the first red circle from Fig. 2(e) in the main text).

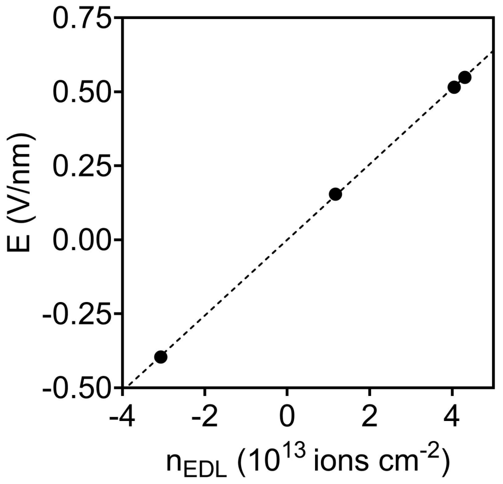

Figure S6: E versus n_{EDL} found from COMSOL modeling shown in Fig.2(e). A linear relationship was found when plotting each time point marked with a red circle in Fig.2(e).

Next, using the Δn_s from Fig. 2(d) and the linear relationship described above, E (V/nm) under AC EDL gating during ER measurements was found. Taking the root mean square of these E values gave E_{RMS} of 0.22 V/nm from AC EDL gating.

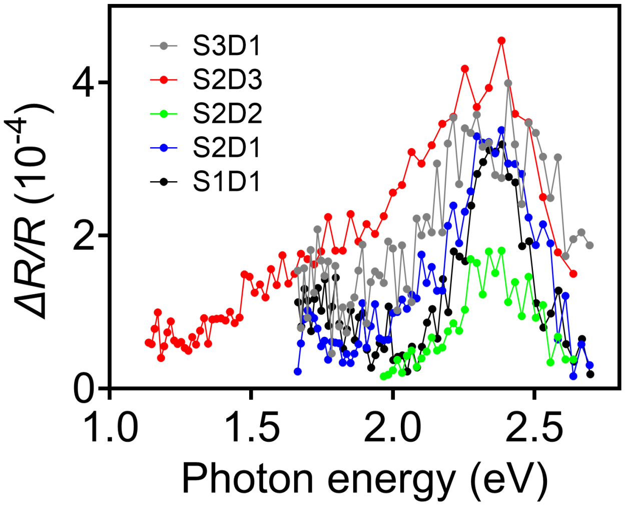

Figure S7: Electroreflectance data for several different 2D Ga/EG electro-optic devices, showing that $\Delta R/R$ has the same general lineshape and magnitude for each device.

5 Optical Transfer Matrix Modeling

The transfer matrix method (TMM)[13] is used to model the absolute and differential reflectivity data based on the ellipsometry model established for EG/bilayer 2D Ga/SiC by Nisi et. al.[14] Fig. S8(a) reproduces the real and imaginary parts of the dielectric function used for the EG and 2D Ga layers in this model and Fig. S8(b) shows the simulated reflectance (R) that results, which is in reasonable agreement with data measured from a wafer-scale 2D Ga/EG sample using a spectrophotometer.

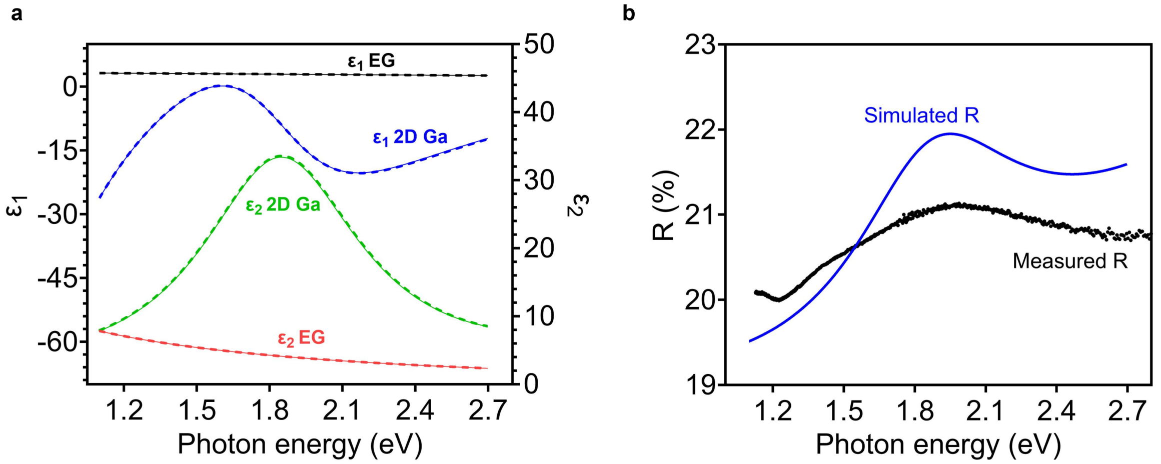

Figure S8: (a) Optical constants used for the 2D Ga and EG layers in the TMM model based on the ellipsometry results of Nisi et al.[14] (b) Measured and simulated absolute reflectance at 8º incidence for a wafer-scale sample of 2D Ga/EG. The peak near $\sim$ 2 eV in both cases originates from the quantum confined interband transition described in the main text.

To model the ER data, we perturbed one or more parameters in the dielectric function model for 2D Ga and EG to compute R_F in the presence of the applied field and then calculated the differential reflectivity, $\Delta R/R = (R_F - R_0)/R_0$ based on the zero-field (unperturbed) reflectance, R_0. Fig. S9 explores the effect of perturbing each parameter in the EG (Drude only) and 2D Ga dielectric function to understand the effect and necessity of each for describing the data. Fig. S9 shows that the Drude contribution in 2D Ga and EG leads to a small change in the differential reflectivity over the NIR to visible spectral range, particularly in the lower energy region ($<$ 2.0 eV) (red line). Substantial changes in

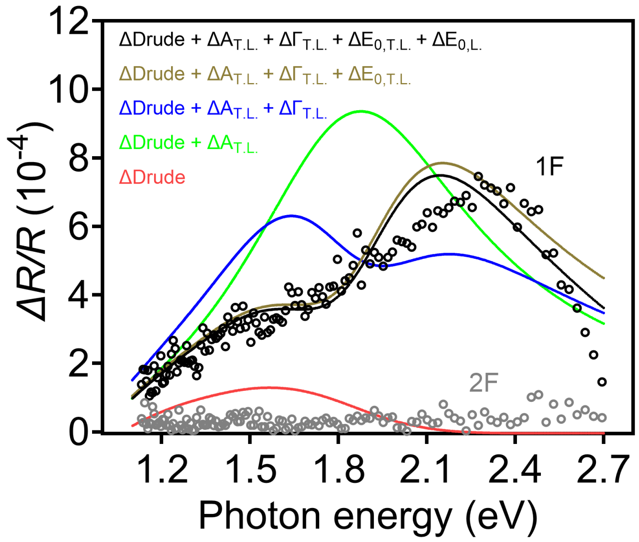

Figure S9: Simulated differential reflectance spectra compared with the ER experimental data for various perturbations of EG (Drude only) and 2D Ga dielectric function parameters, including the magnitude of the Drude contribution (ΔDrude), and the energy, amplitude, and broadening of the Tauc-Lorentz oscillator that describes the interband transition at 1.889 eV ($\Delta E_{0,TL}$, ΔA_{TL}, $\Delta\Gamma_{TL}$, respectively). Shifting the higher energy Lorentz oscillator located at 3.619 eV in the 2D Ga dielectric function model[14] improves the fit of the high energy tail ($\Delta E_{0,L}$, black line), but is unnecessary to capture the main features of the data.

the amplitude and/or broadening of the Tauc-Lorentz oscillator that describes the 1.889 eV interband transition in 2D Ga are similarly ruled out based on their different functional dependence compared to the data (green and blue lines). The best and most robust description of the data is obtained by shifting the energy of this oscillator by 1.8 meV (gold and black lines), which quantifies the magnitude of the Stark shift energy, $\Delta E = -\Delta\boldsymbol{\mu}\cdot\boldsymbol{F}$, in the main text. The black line includes a small shift in the higher energy Lorentz oscillator located at 3.619 eV in the 2D Ga dielectric function model,[14] which improves the fit of the high energy tail but is unnecessary to fit the main features of the data.

References

(1) Kilic, M. S.; Bazant, M. Z. Steric Effects in the Dynamics of Electrolytes at Large Applied Voltages. II. Modified Poission-Nernst-Planck Equations. *Physical Review E* **2007**, *75*, 21503.

(2) Awate, S. S.; Mostek, B.; Kumari, S.; Dong, C.; Robinson, J. A.; Xu, K.; Fullerton-Shirey, S. K. Impact of Large Gate Voltages and Ultrathin Polymer Electrolytes on Carrier Density in Electric-Double-Layer-Gated Two-Dimensional Crystal Transistors. *ACS Applied Materials & Interfaces* **2023**, *15*, 15785–15796.

(3) Woeppel, A.; Xu, K.; Kozhakhmetov, A.; Awate, S.; Robinson, J. A.; Fullerton-Shirey, S. K. Single- versus Dual-Ion Conductors for Electric Double Layer Gating: Finite Element Modeling and Hall-Effect Measurements. *ACS Applied Materials & Interfaces* **2020**, *12*, 40850–40858.

(4) Das, S.; Ghosh, A. Ionic Conductivity and Dielectric Permittivity of PEO-LiClO4 Solid Polymer Electrolyte Plasticized with Propylene Carbonate. *AIP Advances* **2015**, *5*, 027125.

(5) Perdew, J. P.; Burke, K.; Ernzerhof, M. Generalized Gradient Approximation Made Simple. *Phys. Rev. Lett.* **1996**, *77*, 3865–3868.

(6) Neugebauer, J.; Scheffler, M. Adsorbate-substrate and adsorbate-adsorbate interactions of Na and K adlayers on Al(111). *Phys. Rev. B* **1992**, *46*, 16067–16080.

(7) Briggs, N. et al. Atomically thin half-van der Waals metals enabled by confinement heteroepitaxy. *Nature Materials* **2020**, *19*, 637–643.

(8) Li, H.-M.; Xu, K.; Bourdon, B.; Lu, H.; Lin, Y.-C.; Robinson, J. A.; Seabaugh, A. C.; Fullerton-Shirey, S. K. Electric Double Layer Dynamics in Poly(ethylene oxide) $LiClO_4$ on Graphene Transistors. *The Journal of Physical Chemistry C* **2017**, *121*, 16996–17004.

(9) Liang, J.; Xu, K.; Toncini, B.; Bersch, B.; Jariwala, B.; Lin, Y.; Robinson, J.; Fullerton-Shirey, S. K. Impact of Post-Lithography Polymer Residue on the Electrical Characteristics of MoS_2 and WSe_2 Field Effect Transistors. *Advanced Materials Interfaces* **2019**, *6*, 1801321.

(10) Wetherington, M. T.; Turker, F.; Bowen, T.; Vera, A.; Rajabpour, S.; Briggs, N.; Subramanian, S.; Maloney, A.; Robinson, J. A. 2-dimensional polar metals: a low-frequency Raman scattering study. *2D Materials* **2021**, *8*, 041003.

(11) Lee, D. S.; Riedl, C.; Krauss, B.; Von Klitzing, K.; Starke, U.; Smet, J. H. Raman Spectra of Epitaxial Graphene on SiC and of Epitaxial Graphene Transferred to SiO_2. *Nano Letters* **2008**, *8*, 4320–4325.

(12) Xu, K.; Islam, M. M.; Guzman, D.; Seabaugh, A. C.; Strachan, A.; Fullerton-Shirey, S. K. Pulse Dynamics of Electric Double Layer Formation on All-Solid-State Graphene Field-Effect Transistors. *ACS Applied Materials & Interfaces* **2018**, *10*, 43166–43176.

(13) Yeh, P. *Optical waves in layered media*; Wiley: Hoboken, NJ, 2005.

(14) Nisi, K.; Subramanian, S.; He, W.; Ulman, K. A.; El-Sherif, H.; Sigger, F.; Lassaunière, M.; Wetherington, M. T.; Briggs, N.; Gray, J.; Holleitner, A. W.; Bassim, N.; Quek, S. Y.; Robinson, J. A.; Wurstbauer, U. Light–Matter Interaction in Quantum Confined 2D Polar Metals. *Advanced Functional Materials* **2021**, *31*, 2005977.
